# Chronic Rhinosinusitis, *S. aureus* Biofilm and Secreted Products, Inflammatory Responses, and Disease Severity

**DOI:** 10.3390/biomedicines10061362

**Published:** 2022-06-09

**Authors:** Gohar Shaghayegh, Clare Cooksley, Mahnaz Ramezanpour, Peter-John Wormald, Alkis James Psaltis, Sarah Vreugde

**Affiliations:** 1 Adelaide Medical School, Faculty of Health and Medical Sciences, The University of Adelaide, Adelaide 5000, Australia; gohar.shaghayegh@adelaide.edu.au (G.S.); clare.cooksley@adelaide.edu.au (C.C.); mahnaz.ramezanpour@adelaide.edu.au (M.R.); peterj.wormald@adelaide.edu.au (P.-J.W.); alkis.psaltis@adelaide.edu.au (A.J.P.); 2 Department of Surgery-Otolaryngology-Head and Neck Surgery, University of Adelaide, Adelaide 5011, Australia; 3 Central Adelaide Local Health Network, The Queen Elizabeth Hospital, The Basil Hetzel Institute for Translational Health Research, Woodville South 5011, Australia

**Keywords:** chronic rhinosinusitis, *S. aureus* biofilm/virulence factor, inflammatory cells/endotypes

## Abstract

Chronic rhinosinusitis (CRS) is a persistent inflammation of the nasal cavity and paranasal sinuses associated with tissue remodelling, dysfunction of the sinuses’ natural defence mechanisms, and induction of different inflammatory clusters. The etiopathogenesis of CRS remains elusive, and both environmental factors, such as bacterial biofilms and the host’s general condition, are thought to play a role. Bacterial biofilms have significant clinical relevance due to their potential to cause resistance to antimicrobial therapy and host defenses. Despite substantial medical advances, some CRS patients suffer from recalcitrant disease that is unresponsive to medical and surgical treatments. Those patients often have nasal polyps with tissue eosinophilia, *S. aureus*-dominant mucosal biofilm, comorbid asthma, and a severely compromised quality of life. This review aims to summarise the contemporary knowledge of inflammatory cells/pathways in CRS, the role of bacterial biofilm, and their impact on the severity of the disease. Here, an emphasis is placed on *S. aureus* biofilm and its secreted products. A better understanding of these factors might offer important diagnostic and therapeutic perceptions for recalcitrant disease.

## 1. Introduction

### 1.1. Chronic Rhinosinusitis

Chronic rhinosinusitis (CRS) is a persistent inflammation of the nasal cavity and paranasal sinuses for more than 12 weeks. CRS is associated with tissue remodelling, dysfunction of the sinus’s natural defence mechanisms, and induction of different inflammatory clusters [1,2]. Common symptoms of CRS include nasal congestion, rhinorrhoea, sinus pain/pressure, and a reduced sense of smell. Fever, sense of fatigue, ear fullness, foul taste/odour, and disturbance of sleep have also been reported, which lead to a considerable impairment of a person’s quality of life [3]. CRS afflicts up to 10% of the general population, with the greater prevalence reported in developed countries, male patients, the elderly, and asthmatics, thus imposing a considerable direct and indirect burden on the healthcare system and economies globally [1,4]. CRS encompasses a heterogeneous condition in clinical manifestation, histopathology, and therapeutic response, demonstrating a wide spectrum of disease entities with inconsistent pathophysiology [5]. The disease is phenotypically classified into two broad categories, based on the presence (CRSwNP) or absence (CRSsNP) of nasal polyps on nasal endoscopy or computed tomography (CT) imaging [2]. Nasal polyps are noncancerous inflammatory lesions arising from the ethmoid sinus projecting into the nasal airway [6]. Nasal polyps can block the ostiomeatal complex, interfering with paranasal sinus ventilation and drainage [3]. Even though only about 30% of patients with CRS develop nasal polyps, these polyps are linked to higher disease severity and negatively affect patients’ health-related quality of life and productivity [7].

### 1.2. Aetiology of CRS

CRS is a multifactorial disease with numerous systemic, host-related, and environmental triggers contributing to its pathophysiology. Systemic factors comprise genetic disorders, such as cystic fibrosis, autoimmune disease, immunodeficiency disorders, idiopathic conditions such as Samter’s triad, and gastroesophageal reflux disease (GORD). Major host factors include anatomical abnormalities affecting the ostiomeatal complex (such as nasal septal deviation and concha bullosa of the nasal cavities), sinonasal drainage abnormalities, iatrogenic conditions such as postsurgical sinus scarring, and the presence of foreign bodies in the nose. Potential environmental triggers include the existence of bacterial biofilms and its associated infection, fungal infection, allergies, environmental pollutants, and smoking [8]. Despite the high prevalence and substantial health impact of CRS, its aetiopathogenesis has remained incompletely understood.

### 1.3. CRS and Asthma

The prevalence of asthma in CRS patients has been reported to range from 4 to 44% [9,10,11,12,13,14,15]. CRSwNP patients have a much higher comorbidity rate of asthma than CRSsNP patients [16]. CRSwNP and asthma are strongly linked, coexist epidemiologically, clinically, and pathophysiologically [14,17], and influence each other bidirectionally [18]. In Europe, about 20 to 60% of CRSwNP patients have asthma [19,20]. Asthmatic CRSwNP patients have more severe sinonasal symptoms and worse quality of life; their condition is more challenging to treat, both medically and surgically [21]. They are characterised by tissue eosinophilia, upregulation of type 2 cytokines, and high local IgE levels [16,22]. The pathophysiological resemblances of the upper and lower airways have significant implications for both the diagnosis and management of these common comorbidities [21]. In light of the growing understanding of the pathophysiology of concurrent chronic upper and lower airway diseases, the rationale for targeted therapy that focuses on the underlying immune mechanisms of both diseases becomes more compelling.

### 1.4. CRS and Dysbiosis

Originally, the sinuses of healthy individuals were believed to be sterile environments, with CRS emerging as a consequence of bacterial infection [1]. Growing focus on the human microbiome has led to a paradigm shift, and it is now well-understood that diverse bacterial communities colonise healthy sinuses, where they act in symbiosis [23,24].

Research aimed at distinguishing the sinus microbiome in healthy subjects and CRS patients found it to be heterogeneous, with a dramatic decrease in bacterial diversity, as well as a remarkable change in the percentage of specific taxa in CRS patients at post-surgical states, compared to healthy controls [25,26,27]. Feazel et al. revealed that an enhanced relative abundance of *S. aureus* is associated with a diminished total bacterial biodiversity. Their study also demonstrated that elevated exposure to antibiotics is associated with a lower diversity of bacteria. These outcomes suggest that frequent antibiotic use contributes to a constant disturbance of the sinus microbiome, resulting in chronic *S. aureus* colonization [25]. Additionally, the total bacterial burden was reported to be the same in both CRS and control subjects in several studies; however, a noticeable expansion of pathogenic bacteria, particularly *S. aureus* and anaerobes, was revealed in CRS patients. Furthermore, patients with CRSwNP, particularly those with comorbid asthma, possess an increased relative abundance of *S. aureus* [26,28,29].

A long-term analysis, by Koutsourelakis et al., of the microbiome in CRS sufferers revealed that approximately 25% of the sinonasal bacterial taxa remain noticeably constant over time [30]. These stably abundant taxa existed in both the healthy control and CRS subjects. These findings were in line with a more recent study by Paramasivan et al., in which the sinonasal microbiome in a large, multicentre, international cohort of 410 CRS patients and healthy controls was investigated. They showed that the core microbiome within the middle meatus of patients with or without CRS is composed of the genera *Corynebacterium*, *Staphylococcus*, *Streptococcus*, *Moraxella*, and *Haemophilus* [31]. Nevertheless, these main bacterial taxa are accompanied by numerous less abundant taxa that are believed to be responsible for modifying the community dynamics of the microbial niche [30,32]. It is thought that these low-abundant bacteria are crucial for maintaining microbial homeostasis, and the abundance of *S. aureus* becomes clinically evident in a state of chronic inflammation and prolonged use of steroids or antibiotics [33].

Analysing differences in the nasal microbiome within CRS patients is also essential, as nasal polyps might supply niche microenvironments for bacterial colonisation. CRSwNP is strikingly linked to the elevated presence of *S. aureus*, compared to CRSsNP [27,34,35,36,37]. An enhanced abundance of pathogenic bacteria and loss of protective/commensal bacterial strains might be a driving factor in the local immune response observed in CRS sufferers. Interestingly, some bacterial species, including *S. aureus*, have been proposed to have a protective function in the sinus microbiome under normal circumstances; nonetheless, in the context of dysbiosis, their presence is linked with an intense local immune response, as well as disease severity [37]. Hence, an imbalanced sinus microbiome or loss of microbiome diversity appears to be a crucial factor in CRS; however, whether this dysbiosis is a causative or propagative mechanism of inflammation remains controversial. Dysbiosis might contribute to stimulating an inflammatory response, whereas inflammation itself can establish an environment that encourages alterations in the local bacterial residents. A comprehensive analysis of host-microbiome association/interactions, including the analysis of the correlation/effect of microbial metabolites on host immunity, might shed light on the inflammatory responses of CRS patients [38].

### 1.5. Staphylococcus aureus

Diverse areas of the human body, such as the sinonasal mucosa, have their own microbiome comprising numerous microorganisms in low abundance. Any interruption in this balance by a single bacterium’s overpopulation and suppression of other bacterial communities can cause a pathologic state. Common infectious agents of the upper respiratory tract include *S. aureus*, *Haemophilus influenzae*, *Pseudomonas aeruginosa*, and *Moraxella catharralis* [36]. Even though no specific bacterial species has been considered the initial aetiologic factor in CRS, a strong emphasis has been laid on the possible effect of *S. aureus* and its enterotoxins [39]. *S. aureus* is an important human pathogen that is responsible for a broad spectrum of diseases, ranging from minor skin and soft tissue infections to life-threatening conditions, such as endocarditis, osteomyelitis, toxic shock syndrome, and medical device-related infections [40,41]. The asymptomatic carriage of *S. aureus* by humans is the primary natural reservoir, and the anterior nasal mucosa and skin have been thought to be the major ecological niche in more than 50% of the general population [42]. The precise prevalence of *S. aureus* colonisation in human sinuses is not entirely known; however, it has been reported that about 64% of CRSwNP sufferers exhibit nasal cavity colonisation with *S. aureus*, compared to only 33% and 20% of CRSsNP and healthy control subjects, respectively [35]. CRS patients colonised with particular pathogenic strains of *S. aureus* tend to maintain the same strain for a long period of time, despite frequent antibiotic treatments, implying either a resistance to antibacterial agents or presence of a reservoir for bacterial recolonization [43].

### 1.6. Staphylococcal Biofilm

*S. aureus* is notorious as the most frequent agent that causes hospital-acquired infections, and the emergence of antibiotic-resistant strains, such as the methicillin-resistant *S. aureus* (MRSA), challenges healthcare systems worldwide. Strains of *S. aureus* with increased virulence, known as community-acquired MRSA (CA-MRSA), can also pose a threat to healthy individuals [44]. Thus far, no candidate vaccine has proven effective against *S. aureus* infections. This highlights the urgent need to better understand how the bacterium interacts with the host immune system, in order to avoid or prevent protective immunity [45]. The remarkable success of *S. aureus* as a pathogen might be due to the numerous measures it takes to protect itself against the host’s immune system, including the biofilm’s mode of existence. Bacteria present in biofilm express different genes and proteins from their planktonic counterparts [46,47], and they are more resistant to antimicrobial therapy and host defenses [48].

Biofilm forms when planktonic bacteria organise into three-dimensional, multilayered colonies. Biofilm is the ideal mode of existence for an estimated 99% of bacteria. There are several significant differences between the bacteria that establish a biofilm and their planktonic counterparts, with respect to growth dynamics and genetic expression [49,50,51]. The formation of a bacterial biofilm is an intricate process. Primarily, sessile planktonic bacteria attach to a surface and create microcolonies [52]. Initial adherence is shaped by feeble van der Waals forces and might require bacterial flagella [53]. Upregulation in the expression of cell adhesion structures, such as pili, creates robust and permanent interaction [54]. Once bacteria attach to a surface, they initiate the proliferation and secretion of an extracellular polymeric substance (EPS) matrix, consisting mainly of polysaccharides, proteins, and extracellular nucleic acids [55]. The EPS matrix protects the biofilm inhabitants against environmental stress. As the biofilm grows, the concentrations of some signalling molecules, such as cyclic di-guanosine monophosphate (c-di-GMP), increase and lead to alterations in intracellular signalling. This molecule functions to trigger biofilm maturation through the modulation of cell-to-cell adhesion, quorum sensing, metabolic activity, stress response, and the phenotypic conversion from the planktonic form to the biofilm form [56]. Upon biofilm maturation, bacteria within the biofilm transcribe DNA in a synchronised manner, demonstrating the features of a single multicellular organism that can colonise host tissues. Next, biofilms spread by dispersing free-floating planktonic bacteria [57]. These bacteria can attach to distant spots in the host [58].

Due to their highly efficient adaptation mechanisms to changing environments, bacterial biofilms have mastered coordinated defence mechanisms that render them over 1000-fold more resistant to antimicrobial therapy and host defenses than that of their planktonic form [48]. The prevalence of bacterial biofilms in the paranasal sinuses of CRS patients has been reported in about 42–80% of patients, with a notably higher prevalence in CRSwNP [51,59,60,61,62]. The most frequently detected organisms in the composition of CRS biofilms are *S. aureus*, *Haemophilus influenzae*, and *Pseudomonas aeruginosa* [63,64]. Clinically, biofilm-positive CRS sufferers tend to have a higher severity of disease preoperatively, as well as a persistence of postoperative symptoms, infection, and inflammation of the sinonasal mucosa [48,65]. CRS biofilms, particularly those dominated by *S. aureus*, are associated with an unfavourable prognosis and disease recalcitrance, and existing medical therapies fail to eliminate the mucosal bacterial biofilms [66,67,68]. Additionally, a recent study by Cirkovic et al. evaluated the bacterial biofilm production in CRSwNP patients, and *S. aureus* resulted in being a stronger biofilm-producing bacterium, compared to other bacterial species that exist in patients’ polymicrobial flora [69]. On the other hand, bacterial biofilms have also been found in healthy individuals’ sinonasal mucosa, implying that these biofilms might be a normal component of the regular respiratory mucosal blanket [70]. However, with the lack of precise, convincing evidence that the inflammation in CRS is triggered by bacterial biofilms, the presence of inflammation might be considered a secondary consequence of chronic mucosal immune dysfunction and/or mucociliary impairment [5].

### 1.7. Staphylococcal Virulence Factors

**Figure 1:**
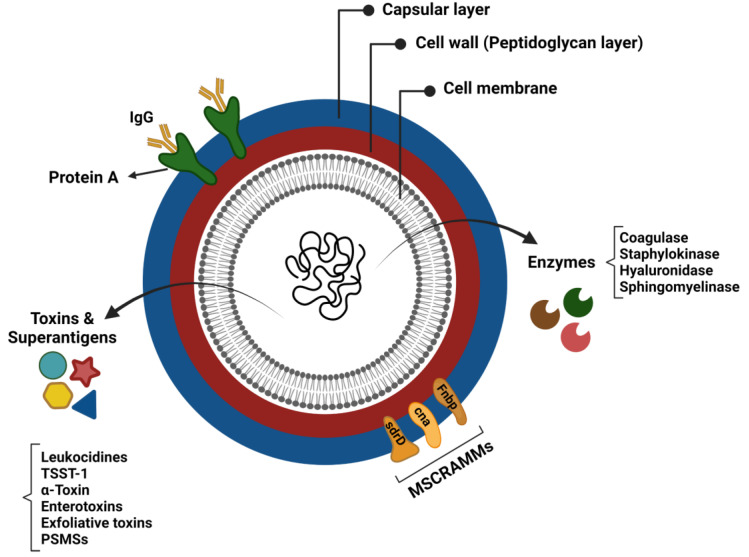
Virulence factors of *Staphylococcus aureus*.

The virulence of *Staphylococcus aureus* is generally considered to be multifactorial, due to the combined activity of an arsenal of virulence determinants that promote tissue adhesion, immune evasion, and host cell damage [71]. These virulence factors consist of structural factors and secreted molecules (exoproteins) (Figure 1) [72]. In addition to *S. aureus* biofilm, a better understanding of each virulence factor’s functions and mechanisms of action is essential for enhancing the prognosis of patients suffering from CRS.

#### 1.7.1. Structural Factors

##### Adherence Factors (Adhesins)

Multiple adhesin proteins mediate the *S. aureus* attachment to the host cell surface and initiate colonisation. *S. aureus* adhesins comprise proteins linked to cells via peptidoglycans that specifically bind to plasma or extracellular matrix components and are collectively referred to as microbial surface components recognising adhesive matrix molecules (MSCRAMMs) [73]. Typical members of the MSCRAMM family are staphylococcal protein A (SpA; encoding by *spa* gene), fibronectin-binding proteins A and B (FnbpA and FnbpB; encoding by the *fnbA* and *fnbB* loci), the serine–aspartate repeat proteins (encoding by *sdrC*, *sdrD*, and *sdrE* genes), collagen-binding protein, and clumping factor (Clf) A and B proteins [72]. These adhesins are closely related to the pathogenicity of staphylococci, since their adherence to the extracellular matrix or plasma proteins is a crucial step in the formation of biofilm and invasion of host cells [74,75]. For instance, spA effectively inhibits opsonisation and the subsequent phagocytosis by attaching to, and neutralising the activity of, the Fc domain of the IgG antibodies. It is, indeed, a B cell superantigen-inducing proliferative expansion and programmed cell death [76]. 

##### Polysaccharide Capsule

*S. aureus* polysaccharide capsule is a virulence factor that can protect the bacterium from complement binding and the subsequent phagocytic killing by neutrophils [77,78]. Most clinically important *S. aureus* isolates possess a type 5 or 8 polysaccharide capsule [79]. These polysaccharide capsules can also act as physical barriers to prevent bacteriophage infection [80,81]. Bacteriophages can integrate their DNA into the *S. aureus* genome and infect them with highly virulent genes, such as *lukF.PV* and *sea* [82], thus influencing *S. aureus* pathogenicity and CRS severity [83].

#### 1.7.2. Exoproteins

Almost all *S. aureus* strains secrete a group of exoproteins, such as exotoxins and enzymes, converting host tissues into nutrients required for bacterial growth [84]. Despite the broad range of *S. aureus* exotoxins, many members have remained uncharacterised for their role in staphylococcal pathology [85]. Human-specific exotoxins are classified as pore-forming toxins, enzymatic toxins, and superantigens.

##### Pore-Forming and Enzymatic Toxins

*S. aureus α*-toxin and bi-component leukocidins are two important pore-forming toxins that act by initial recognition of a receptor determinant on the surface of the target cells, followed by oligomerisation and pore formation [85], which exaggerate the host inflammatory response by inducing the expression of proinflammatory cytokines and lysing inflammatory cells to release additional inflammatory mediators [72].

α-*Toxin: S. aureus* α-toxin (hemolysin-α) is the major cytotoxic agent released by the organism, and it represents the first pore-forming bacterial exotoxin to be identified [86]. The formation of pores on susceptible host cell membranes alters the ion gradients, compromises membrane integrity, activates stress signalling pathways, and causes cell death [87]. α-toxin exerts its effects by interacting with the specific host component ADAM10 [88]. ADAM10 is a cellular metalloprotease responsible for various functions, including E-cadherin shedding and endothelial permeability [89,90]. The α-toxin of *S. aureus* plays a critical role in the pathogenesis of staphylococcal infection, as mutant strains lacking hla have reduced virulence in invasive disease models [91]. α-toxin at low concentrations forms heptameric pores on the surface of cells. Monovalent ions are exchanged through these pores, resulting in DNA fragmentation and apoptosis [92]. α-toxin at high concentrations is absorbed nonspecifically into lipid bilayers and forms large, Ca^2+^-permissive pores. The uncontrolled Ca^2+^ influx causes massive necrosis and other secondary cellular reactions [93]. A variety of human cell types are affected by this toxin, including epithelial cells, endothelial cells, T cells, monocytes, macrophages, and platelets [94]. The ability of the α-toxin to contribute to virulence makes it an ideal target for developing anti-toxin treatments against *S. aureus*.

Leukocidins: Leukocidins, as one of the pore-forming toxins of *S. aureus*, can damage the cell membranes of the host, either by degrading the inter-cellular connections or by modulating the immune responses [44]. Four bi-component leukocidins that are structurally similar to Hla include γ-hemolysin (HlgA, HlgC, and HlgB), leukocidin ED (LukE and LukD), leukocidin AB/GH (LukAB/LukGH), and Panton–Valentine leukocidin (PVL) [95]. These leukocidins lyse cells of the leukocytic lineage and are known to kill neutrophils, while only γ-hemolysin and LukED have demonstrated lytic activity against red blood cells [95,96,97,98]. Panton–Valentine leukocidins (PVL) possess a 100-fold higher leukocytotic activity than the others [44]. Leukocidins’ leukocytotic activity is determined by receptor interaction. The LukED receptor on immune cells is CCR5, while C5aR, C5L2, and CD11b are the receptors for PVL and LukAB [99,100,101]. All PVL-producing isolates produce ‘S’ and ‘F’ subunits, and all PVL genes (*lukS-PV* and *lukF-PV*) are encoded in several bacteriophages carrying the Sa2 integrase [102,103]. The contribution of this leukocidin in *S. aureus* virulence is not yet conclusively proven; however, it is strongly associated with community-acquired MRSA strains, particularly those causing pneumonia and skin and soft tissue infections [95]. This linkage to virulent strains indicates its ability to cause deadly infections in healthy individuals [104]. A study conducted by Gillet et al. showed that pneumonia associated with PVL-positive *S. aureus* was more lethal than that associated with PVL-negative *S. aureus*. Autopsy of the patients revealed that those with PVL-positive *S. aureus* had ulcerated and hemorrhagic lungs, suggesting an extreme inflammation [105]. Infection with PVL-positive strains appears to predict the clinical outcome for skin and soft tissue disease, and the risks of requiring surgical intervention seem to be higher for patients with PVL-positive skin and soft tissue disease [106]. PVL-positive *S. aureus* strains are becoming more common, and some of these strains are MRSA with limited treatment options. Thus, new insights into its role and contribution to CRS pathophysiology might enable the development of new effective antimicrobial strategies [100].

β-hemolysin: The role of β-hemolysin (sphingomyelinase) in disease is unclear. These toxins have been reported to have cytotoxic effects on human keratinocytes, polymorphonuclear leukocytes, monocytes, and T lymphocytes, as well as inhibiting the expression of IL-8 by endothelial cells. These lead to *S. aureus* phagosome escape and biofilm development [107,108,109,110]. This toxin has also been shown to be important for the pathogenicity of *S. aureus.* Previous studies have shown that *S. aureus* mutants lacking hlb are less virulent in pneumonia and murine ear skin infections [107]. The pathogenicity of a mutant strain expressing biofilm formation-deficient β-hemolysins was also found to be reduced in a rabbit endocarditis model [111].

Phenol-Soluble Modulins: Phenol-soluble modulins (PSMs), as one of the most important virulence factors in *S. aureus*, are involved in various pathological processes, including the lysis of red and white blood cells, induction of inflammatory responses, and antimicrobial activities [112,113,114]. PSMs have also been associated with the structuring and detachment of biofilms [115,116]. *S. aureus* produces a variety of PSMs, including PSMα, PSMβ, and δ-toxin, which are pore-forming toxins, and attaches to the cytoplasmic membrane non-specifically, causing membrane disintegration [112]. It has been shown that PSMα peptides influence the ability of community-associated MRSA (CA-MRSA) to cause skin infection and bacteraemia [117]. Thus, PSMs might be a good target for developing anti-staphylococcal treatments, as eliminating their cytolytic and pro-inflammatory activities would likely reduce their potency against host cells, as well as their contribution to disease progression [118].

Exfoliative Toxins: Exfoliative toxins (ETs), also known as epidermolytic toxins, are highly specific serine proteases secreted by *S. aureus*. These proteases enzymatically hydrolyse desmosome cadherins in the superficial layers of the skin [119,120]. ETs are exotoxins that cleave keratinocytes’ junctions and cell-to-cell adhesion in the host’s epidermis, resulting in skin peeling and blistering [121]. ETA and ETB have received the most attention among other ETs, due to their link to staphylococcal scalded skin syndrome (SSSS) [120]. ETs have long been known to possess mitogenic effects on T lymphocytes [122]; however, whether they should be considered superantigens remains controversial.

##### Superantigens

*S. aureus* releases different exotoxins that are capable of functioning as superantigens (SAgs) [71]. Staphylococcal SAgs include the toxic shock syndrome toxin 1 (TSST-1), staphylococcal enterotoxins (SEs), and staphylococcal superantigen-like (SSL) toxins [44]. SAgs are highly mitogenic exotoxins that trigger an enormously powerful stimulatory activity for T lymphocytes. In contrast to conventional peptides, staphylococcal SAgs are introduced by human leukocyte antigen molecules (HLA-α) to the variable β-chain of the T cell receptor (TcRVβ) [123]. They bind to the outer surface of the conventional peptide-binding groove, resulting in an excessive (up to 30%) and uncoordinated T cell reaction with concurrent B cell proliferation, in contrast to the classical HLA presentation, which stimulates only 0.01% of the T cell population [124]. In patients with CRS, this generalised excessive stimulation of the T cells leads to a substantial release of T cell mediators and pro-inflammatory cytokines [124,125], thus intensifying the type 2 inflammatory response. This T cell activation also results in granulocyte migration and survival and is associated with elevated production of IgE, IgA, and IgG/IgG4 antibodies [126,127]. Staphylococcal enterotoxins-IgE (SE-IgE) within the polyp tissue and serum of CRS patients is associated with severe upper and lower airway inflammation, manifesting as nasal polyps with comorbid or severe asthma [128]. 

### 1.8. Immune Response in CRS

CRS is an inflammatory disease, and various cells, including epithelial cells, endothelial cells, fibroblasts, mast cells, neutrophils, eosinophils, dendritic cells, T cells, and B cells, have been demonstrated to be involved in its immune-inflammatory network. These cells normally exert their effect by secreting various mediators, such as cytokines, chemokines, antibodies, and eicosanoids [129].

Failure in the protective measures of the upper respiratory system can lead to the persistence of microbial colonisation, secretion of cytokines and chemokines, recruitment of various immune cells, and activation of inflammatory pathways [130]. The innate immune system represents the first line of defence against inhaled pathogens and foreign substances, and it relies on a large family of pattern recognition receptors (PRRs), which identify distinct evolutionarily conserved structures on pathogens, termed pathogen-associated molecular patterns (PAMPs). The most widely studied PRRs are known as toll-like receptors (TLRs). The binding of PAMPs, including foreign nucleic acids, chemical products, or physical structures, to the ligand-domain of TLRs triggers downstream signal transduction, thus leading to secretion of proinflammatory molecules, such as chemokines and cytokines, which boost the antigen presentation, induction of co-stimulatory molecules of dendritic cells, recruitment of immune cells, and finally, orchestrate the early host response to infection [131]. 

#### 1.8.1. Innate Immune Response

The upper respiratory tract possesses several defence mechanisms against invading pathogens, allergens, and irritants that are apparently overcome in CRS. As the first line of defense, the sinonasal epithelium is in constant contact with inhaled pathogens and harmful particulates. The interaction between epithelial cells and pathogens encompasses an intricate collection of innate and adaptive immune pathways at the mucosal layer. The sinonasal epithelium, besides providing a physical barrier and preserving mucociliary clearance, regulates the innate immune response by releasing various cytokines and chemokines [132]. The significance of the epithelial barrier and its function in CRS has been stated in several studies [133,134]. A damaged airway epithelium has been reported in several chronic airway disorders, such as acute and chronic rhinosinusitis, as well as asthma, and is linked to the severity and chronicity of the induced inflammation [133,135,136].

Natural killer cells: Natural killer (NK) cells, as one of the essential components of innate immunity, have important roles in regulating immune responses by releasing cytokines and inducing cytotoxicity against infected cells. The involvement of NK cells in the pathophysiology of CRS has not yet been entirely elucidated [137]. However, NK cells with impaired effector functions have been shown, particularly in CRS patients with peripheral blood eosinophilia, asthma, and disease recalcitrance. NK cells also exhibit reduced degranulation capacity and diminished release of IFN-γ and TNF-α in those patients [138]. NK cells regulate the activation and apoptosis of inflammatory cells such as eosinophils and neutrophils [139,140]. In eosinophilic CRS patients, peripheral blood NK cell-mediated eosinophil apoptosis is significantly reduced, compared to healthy controls. Additionally, sinus tissue NKp46+ NK cell counts is inversely associated with tissue eosinophil counts. NK cell reduction in an eosinophilic mouse model of CRS exacerbates sinonasal eosinophilic inflammation and declines apoptotic eosinophils in the sinonasal tissue. Interestingly, prostaglandin D_2_, which has been shown to suppress cytotoxicity and IFN-γ and TNF-α production in NK cells, increases in CRS patients [141]. Therefore, eosinophilic inflammation in CRS is strongly associated with prostaglandin D_2_ dysregulation and the subsequent impairment of NK cell-mediated eosinophil apoptosis. The inhibition of prostaglandin D_2_ and restoring of NK cell activity might be possible treatment strategies in eosinophilic CRS [142].

Eosinophils, Mast Cells, and Basophils: Eosinophils are circulating granulocytic leukocytes and one of the immune system components that express various surface receptors. Some of these receptors are common to different innate immune cells, and some are unique, such as the IL-5R, CCR3, and Siglec proteins [143]. IL-5R binds to T cell cytokine IL-5, which is critical for eosinophil survival, growth, recruitment, and activation [144]. Stimulated eosinophils elicit their bactericidal response by the extracellular release of their cytoplasmic granules. The important eosinophil granule proteins include the eosinophil cationic protein (ECP), major basic proteins (MBP1 and MBP2), eosinophil-derived neurotoxin (EDN), and eosinophil peroxidase (EPX). These proteins activate other immune cells and are major contributors to the toxicity to microorganisms through the generation of reactive oxygen species (ROS) and direct killing of bacteria [145]. The proinflammatory mediators originating from eosinophils are major contributors to airway epithelial damage, hypersensitivity, mucus secretion, and airway remodelling, which are the hallmarks of chronic respiratory and sinonasal inflammation [146]. For instance, eosinophils exert their cytotoxic effect by releasing major basic proteins (MBPs), which leads to epithelial damage [147]. RNA sequencing of stimulated human nasal epithelial cells with EDN has also demonstrated an increased expression of MMP-9 [148], as a contributing factor to tissue remodelling [149]. Eosinophils can also release extracellular traps containing nuclear DNA through cytolytic extracellular trap cell death, thus contributing to CRS pathogenesis, particularly in CRSwNP with *S. aureus* colonization [150,151].

Eosinophils are one of the major hallmarks of type 2 inflammatory patterns in CRSwNP patients in western societies [130]. Eosinophilic nasal polyps were found to be associated with severe sinus inflammation on both CT scans and nasal endoscopies, compared to non-eosinophilic nasal polyps [152]. Tissue eosinophilia increases the likelihood of recurrent disease and comorbid asthma in CRSwNP patients, suggesting that eosinophils contribute substantially to the pathology of CRSwNP [153,154]. However, the exact role of eosinophils in airway disease, as well as their involvement in inflammation, is not entirely elucidated.

Apart from eosinophils, other type-2 innate inflammatory cells, such as mast cells (MCs) and basophils, are elevated in CRSwNP, compared to healthy controls [155,156]. MCs have been found to be higher in nasal polyps of eosinophilic CRS patients, compared to nasal polyps of non-eosinophilic CRS patients, indicating the positive correlation between eosinophilia and MCs activation in CRS [157,158]. MCs and basophils generate inflammatory mediators and toxic granule proteins, maintaining the inflammatory response and promoting sinonasal mucosal injury. A distinct subclass of MCs was detected in nasal polyp glandular epithelial cells. They are capable of producing tryptase, carboxypeptidase A3, and chymase. The latter is a common inducer of mucus, and it is postulated that these specific MCs might play a central role in mucus hypersecretion, which is frequently observed in CRSwNP patients [156]. Nasal polyps infiltrated by MCs express higher T cell immunoglobulin and mucin domain protein 3 (TIM-3), a receptor that promotes MCs activation and cytokine production [159]. Despite the higher expression of TIM-3 in the epithelium than in the stroma of nasal polyps, the infiltration of MCs into the stromal layer is associated with the severity of CRSwNP and resistance to medical and surgical treatments [160].

Additionally, a small study in patients with CRSwNP has claimed that MCs might function as a reservoir for *S. aureus* and contribute to the disease chronicity in certain patients [161]. It is well-known that MCs are activated by local IgE and secrete large amounts of type 2 cytokines, thus facilitating type 2 responses and eosinophilic inflammation in CRSwNP patients. However, the disease-specific mechanisms of MCs-triggering in CRSwNP, aside from IgE, are poorly understood [162]. An endotoxin-releasing strain of *S. aureus* has been reported to promote the internalisation of *S. aureus* into MCs through phagocytosis, leading to intracellular *S. aureus* proliferation and expansion and MCs rupture. This eventually results in proinflammatory mediators and cytokines release [163].

Neutrophils: Neutrophils are considered important components of the innate immune system, due to their numerous bacterial killing and sequestration activities. They apply three means for directly attacking microorganisms: phagocytosis, producing neutrophil extracellular traps (NETs), and the release of soluble antimicrobials from their primary (azurophilic) and secondary (specific) granules [164]. Neutrophils are categorised as either N1 or N2. N1 serves as a pro-inflammatory neutrophil, secreting pro-inflammatory cytokines and chemokines, such as TNF, IL-1β, CCL3, CCL5, IL-6, and IL-12, whilst N2 functions as an anti-inflammatory cell with strong immunosuppressive activity [165].

Multiple studies reported the increased proteolytic activity of elastase and cathepsin G, two granule proteins secreted by activated neutrophils, in the tissue of patients with CRSwNP [166,167]. Elastase and cathepsin G, once secreted, are less efficient at microbial killing, but they are highly effective at stimulating the production of cytokines within the IL-1 family, such as IL-1β, IL-33, and IL-36γ [168]. IL-36γ promotes tissue neutrophil secretion of IL-8 and IL-17, reinforcing a positive feedback loop on their recruitment [167,169,170,171]. Elastin, collagen, and fibronectin, as the main components of the extracellular matrix, are subject to neutrophil proteolytic degradation, leading to tissue remodelling [172]. Serine proteases of neutrophils negatively affect the nasal epithelial barrier integrity, while elastase is involved in goblet cell metaplasia and mucus production [173,174,175]. Furthermore, patients with CRSsNP and CRSwNP have shown increased levels of neutrophil extracellular traps (NETs) in their secretions, as well as at subepithelial sites in their tissue [151,166,176,177]. Although recent evidence highlights the significance of neutrophils in the pathogenesis of CRS, various aspects of this inflammation, including the relationship between the microbiome or bacterial biofilms and neutrophilic inflammation in CRS, remain unknown.

Macrophages: Macrophages are generated in the bone marrow by progenitor cells. Tissue macrophages are differentiated from circulating monocytes when those enter the tissue. Tissue macrophages function as phagocytes ingesting pathogens and are the leading players in tissue remodelling after damage [178,179]. Macrophages are subdivided into two distinct phenotypes, based on their function. M1 macrophages, known as classically activated macrophages, are highly phagocytic and mediate host defense and anti-tumour immunity. M1 macrophages are induced by lipopolysaccharides (LPS) and IFN-γ, thus releasing inflammatory cytokines and contributing to Th1 responses [180]. Conversely, wound-healing macrophages, or alternatively activated M2 macrophages, suppress immune responses with the regulation of wound healing [181]. M2 macrophages are induced by Th2 cytokines (IL-4 and IL-13), propagate the Th2 response, and are associated with allergic disease [182,183]. The increased number of macrophages in CRSwNP has been reported in several studies. Krysko et al. have reported that M2 macrophages are substantially elevated in CRSwNP, and Th2 inflammatory markers are positively correlated with the quantities of macrophages [184]. CCL23, a chemokine involved in the recruitment of macrophages, has also been demonstrated to be considerably up-regulated in CRSwNP tissue, suggesting that macrophages might be recruited into nasal polyps by CCL23, and the presence of Th2 cytokines subsequently results in skewing toward the M2 phenotype [185]. In tissue remodelling by Th2-induced coagulopathy, M2 macrophages, alternatively activated by Th2 cytokines [184], secrete coagulation factor XIIIa, which works enzymatically and contributes to creating a tight tetrameric complex (FXIIIA2B2), the cross-linking process of fibrin, resulting in oedematous remodelling patterns in nasal polyps [186].

Innate Lymphoid Cells: Innate lymphoid cells (ILCs) are the most recently identified population of immune cells. ILCs are a subclass of innate immune cells, which are derived from common lymphoid progenitors lacking lineage markers and rearranged antigen-specific receptors. These cells contribute to immune responses by producing different inflammatory cytokines [187,188]. Three ILC subgroups (ILC1, ILC2, and ILC3) exist, and they are characterised by their profile of secreted cytokines and transcription factors guiding their differentiation. These subgroups parallel the T helper subclasses, such that IFN-γ is the key cytokine of ILC1 and Th1 cells; ILC2 and Th2 cells are characterised by IL-5, IL-13, and IL-4 secretion; and ILC3 produces IL-17 and IL-22 [189]. ILCs and T cell subsets display striking similarities in transcription factors, as ILC1, ILC2, and ILC3 are driven by T-bet, GATA-3, and RORγt, respectively [190]. ILCs can instantly respond to environmental stresses, whereas T and B cells require longer to generate an immunologic response. Despite the considerable overlap in the cytokine patterns between ILCs and T helper cells, it remains uncertain whether ILCs have independent responsibilities or are redundant in the immune system [191]. The activation of ILC2 is initiated by several epithelial cell-derived inflammatory mediators, such as IL-25, IL-33, thymic stromal lymphopoietin (TSLP), and lipid mediators [192]. ILCs, in addition to having a critical role in the innate immune response via producing various cytokines against microorganisms and other environmental threats, in homeostasis, operate in the repair and remodelling of tissues [193]. ILCs in human tissue and peripheral blood have been detected in several studies. Although no evidence has been shown of ILC1 and ILC3’s contribution to CRS pathogenesis, ILC2 have been particularly involved in CRSwNP because they are the main source of Th2 cytokines. ILC2 is substantially increased in eosinophilic nasal polyps, implying a role in CRSwNP pathogenesis [194,195,196,197].

#### 1.8.2. Adaptive Immune Response

Helper T cells: T and B cells are effectors of adaptive immunity that have various capabilities in orchestrating inflammation. B cells and T cells possess genetically rearranged and highly diverse antigen receptors that impart specificity to these cells. The participation of CD4+ T cells in the pathogenesis of CRS is well-established, and CD4+ T cells possess various T helper subclasses, such as Th1, Th2, Th9, Th17, and Th22, that are responsible for producing specific cytokines [198,199]. The corresponding cytokine for Th1 cells is IFN-γ, whilst Th2 cells produce IL-4, IL-5, and IL-13. IL-9 is produced by Th9 cells, and IL-17a and IL-22 are released by Th17 and Th22 cells, respectively. These cytokines are not entirely restricted to a specific subgroup in humans, but their ratio determines the ultimate consequence of immune activation [200]. The most prominent T helper cell groups in CRS are Th1, Th2, and Th17. Diverse T cell polarisations affect the choice of treatment strategies for CRS [19,201].

Regulatory T cells (Tregs), as a specialised subpopulation of helper T cells, suppress the immune response, maintain homeostasis and self-tolerance, and prevent autoimmune disease. Tregs are immunosuppressive cells that normally suppress the induction and proliferation of effector T cells [166]. Deficient recruitment of Tregs in CRSwNP has been reported in several studies [166,202], which has been claimed to be due to the impaired migratory function of these cells. The attenuated migration capability of Tregs in CRSwNP results in their decreased nasal mucosal infiltration, which ultimately contributes to inflammation [203]. In addition, other studies have indicated a defective secretion of TGF-β from Tregs in CRSwNP patients [19,204]. The cytokine signalling 3 (SOCS3) protein responsible for the reduced expression of FOXP3 (as a critical regulator of Tregs) has also been demonstrated to be upregulated in Tregs of CRSwNP, supporting the significant involvement of Tregs in CRS pathogenesis [205]. Transcription factor analysis of CRS has also shown a significant up-regulation of GATA-3 and down-regulation of FOXP3 in CRSwNP, compared to CRSsNP [206].

Cytotoxic T cells: CD8+ T cells are another subpopulation of adaptive immune lymphocytes. CD8+ lymphocytes, when activated, can become cytotoxic T (Tc) lymphocytes. The common role of Tc lymphocytes is to destroy and eradicate intracellular pathogen-invaded and tumour-transformed cells through their cytotoxic activity [199,207]. The cytolytic pathway of Tc lymphocytes depends largely on the perforin- and granzyme B-mediated induction of target cell apoptosis or lysis [207,208,209]. Furthermore, growing evidence has recently indicated that CD8+ T cells might also control pathologic processes, such as autoimmune and allergic diseases, through mechanisms beyond their typical cytotoxic activity. Like CD4+ T helper cells (Th), CD8+ cytotoxic T lymphocytes can differentiate into at least five effector subsets with diverse cytokine-producing phenotypes: IFN-γ+ Tc1, IL-4+ Tc2, IL-9+ Tc9, IL-17A+ Tc17, and CD8+ regulatory T cells. The infiltration of these Tc subsets to the site of inflammation is probably mediated by a complex interaction of cytokines, chemokines, and adhesion molecules [210]. Although an enhanced number of CD8+ T cells in sinonasal mucosa of CRS patients has been reported in several studies [211,212,213,214], the functional significance of these cells in the pathogenesis of CRS remains unclear.

B Cells: B cells contribute considerably to the ongoing sinonasal inflammation observed in CRS patients [215]. B cells can be activated in different ways. Once activated, naive B cells develop into antibody-secreting plasmablasts, plasma cells, or memory B cells [216,217]. The first and rapid B cell antibody responses are dominated by plasmablasts, which are situated in the peripheral immune organs. These plasmablasts experience clonal expansion, which results in the generation of large quantities of terminally differentiated short-lived antibody-producing plasmablasts [218]. These plasmablasts can further differentiate into long-lived plasma cells that maintain long-term antibodies production, and they possess enhanced survival and circulation capacity throughout the body [219]. Regulatory B cells (Bregs) can also be induced and exert immunosuppressive properties by secreting IL 10, IL 17, IL 35, and TGFβ, thereby modulating T cell responses [220,221,222].

The production of IgA and IgE from B-lineage cells is known to be essential to allergy. Although the antigen specificity of the antibodies in nasal polyps of CRS patients remains largely undefined, there is evidence that some of the antibodies are autoreactive [223,224], and some of them, especially among the IgE antibodies, are specific to *S. aureus*-derived enterotoxins [22,225]. Interestingly, IgE antibodies against *S. aureus* and its enterotoxins have shown potential to be employed as biomarkers of disease severity [16,226]. IL-13, as a Th2 inflammatory marker, is a critical inducer of IgE class switch recombination and IgE production in B cells [215]. Nasal polyp-localised polyclonal IgE seems to be functional, due to the induction of histamine release from tissue extracts exposed to antigens [227]. 

The overexpression of B cell-activating factor of the TNF family (BAFF) in nasal polyps of CRS patients might be another potential mechanism for local activation of B cells in these patients [228,229]. The significance of BAFF, as well as its contribution to B cell activation and differentiation to plasma cells, has been reported in several studies [215]. Furthermore, Two B cell chemokines, CXCL12 and CXCL13, with a significant increase in nasal polyps, are suggested to contribute to the initial recruitment of B-lineage cells [230].

Memory B cells are long-lived cells that respond faster and with a more robust antibody response on the second encounter with the same antigen to which the naive B cells were exposed [231]. In nasal polyps of CRSwNP patients, the numbers of naïve B cells and activated plasma cells are increased, compared to mucosa from CRSsNP patients or healthy controls [126,229]. Another study carried out by Miljkovic et al. has reported a significant increase in mucosal B cell numbers, including naive B cells, plasmablasts, and memory B cells in CRSwNP patients versus controls [232]. Furthermore, a study examining the biomarkers of inflammation and antibody isotypes in CRS has demonstrated a significant elevation in Th2 inflammation markers, considerable increase in B and plasma cells, and notable increase in IgE levels of CRSwNP patients [233]. 

A subclass of B cells has recently been identified to exert immune-suppressive characteristics by direct interaction with CD4+ T helper cells and IL-10 and TGF-β release. This subgroup is known as regulatory B cells (Bregs) and contributes to stimulating and preserving immune tolerance in allergic diseases and autoimmunity [234]. The use of genetically modified mice that lack B cells [235], and more specifically, IL-10-producing B cells [221], has been shown to impair Bregs development and function, thus leading to Tregs deficiency, overactivation of pro-inflammatory T cells, and chronic inflammation [236]. This implies that these cells could be targeted therapeutically for mitigating a wide variety of immune-mediated inflammatory conditions [234]. Bregs have also demonstrated critical functions in the allergen-specific immunotherapy of allergic rhinitis; nevertheless, their precise roles in CRS pathogenesis remain elusive [234]. 

### 1.9. CRS Inflammatory Endotypes

Endotypes of CRS are commonly characterised based on underlying immune responses and cellular differentiation, specifically CD4+ T helper (Th) cells, CD8+ cytotoxic T (Tc) lymphocytes, and ILCs, which regulate the expression of various chemokines and cytokines [16,129,201]. The type 1 response is mainly associated with CRSsNP and is predominately defined by the increased neutrophils linked to myeloperoxidase and elevated secretion of IFN-γ, IL-2, and TNF-α from ILC1, Tc1, and Th1 cells [237,238]. Type 2 inflammation is associated with CRSwNP in Caucasian patients, and it is primarily characterised by high levels of eosinophils and increased quantities of IL-4, IL-5, and IL-13 from ILC2, Tc2, and Th2 cells, as well as large amounts of eosinophil cationic protein (ECP). Total IgE and *S. aureus* enterotoxin-specific IgE are also increased in patients with CRSwNP [16,193,239]. Finally, type 3 immune response, which has recently been reported to be dominant in Asian patients with CRSwNP, is associated with elevated IL-17 and IL-22 cytokines from ILC3, Tc17 cells, and Th17 cells [128,193].

#### 1.9.1. Mechanism of Type-2 Inflammation

**Figure 2:**
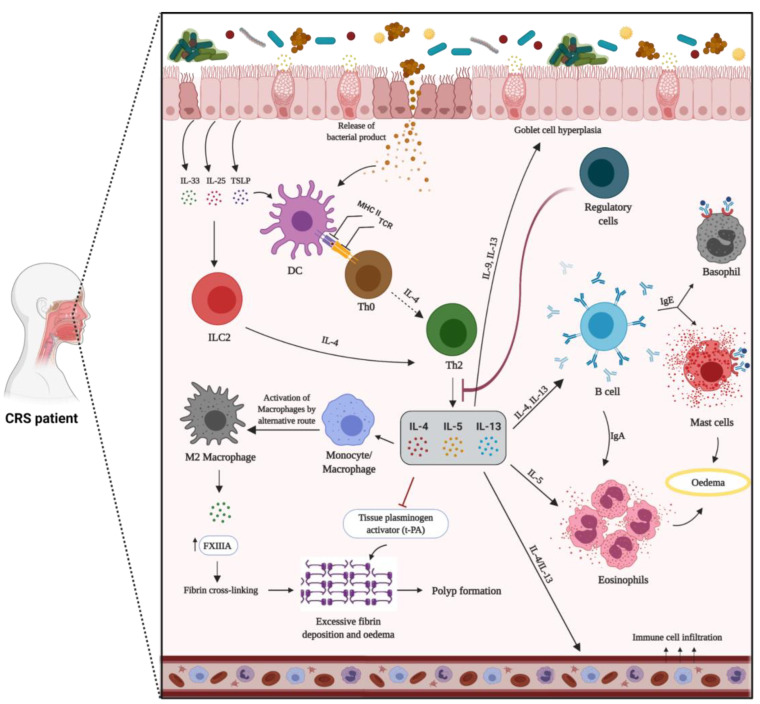
Mechanism of type 2 inflammation in patients with chronic rhinosinusitis with nasal polyps (CRSwNP). The dysregulated epithelial barrier results in an enhanced exposure to inhaled pathogens and allergens. In response to environmental stimuli, epithelial cells secrete inflammatory mediators, such as TSLP, IL-25, and IL-33, thus promoting the development of the type 2 immune response. In this pathway, naïve T cells (Th0) differentiate into Th2 cells, leading to the secretion of IL-4, IL-5, and IL-13. Innate immune cells, including ILC2, eosinophils, and mast cells, are activated and release type 2 cytokines that further perpetuate the ongoing inflammatory response and specific granule proteins that contribute to tissue injury. B cells and activated plasma cells are also increased, thus contributing to the enhanced local generation of antibodies. Type 2 cytokines can result in decreased tissue plasminogen activator and increased FXIIIA levels, which, in a state of an increased vascular leak, can lead to enhanced fibrin cross-linking and deposition within nasal polyps. In a type 2 inflammatory pathway with a general lack of regulatory T cells function, IL-5 triggers eosinophilia, and IL-4 and IL-13 are responsible for local IgE production by B cells. Furthermore, IL-4 and IL-13 lead to goblet cells hyperplasia and excess mucus production.

Type 2 inflammation is the most common endotype in Caucasian patients with eosinophilic CRS and CRSwNP in Western countries. TSLP, IL-25, IL-31, and IL-33 secreted from epithelial cells are known to induce or boost type 2-driven inflammation by activating ILC2s and stimulating the maturation of Th2 cells. In turn, Th2 cells, ILC2s, and Tc2 cells orchestrate eosinophilic inflammation via the production of type 2 cytokines, IL-5, IL-13, and IL-4 [240,241]. It has been shown that IL-5 is significantly higher in CRSwNP, compared to CRSsNP and healthy controls, and might be a useful biomarker to predict type 2 inflammation in patients with CRSwNP [242]. IL-4 and IL-13 have overlapping functions due to their shared receptor affinity for IL-4Ra, and both boost adaptive Th2 responses through the stimulation of B cells and local IgE generation. The increased production of IgE results in mast cell activation, particularly in nasal polyps [227]. Th2 inflammation can also induce monocyte/macrophage differentiation into M2 macrophages, producing coagulation factor XIII-A that stimulates excessive fibrin formation [186]. The tissue plasminogen activator (t-PA) has also shown diminished expression in Th2 inflammation [243]. The altered expression of these factors might clarify the mechanism of water retention and oedema creation in nasal polyps of CRS patients (Figure 2).

*S. aureus* is found in about 60% of CRS patients with eosinophilic inflammation and nasal polyps [35]. It has been reported that *S. aureus* can further skew the immune response to type 2 by regulating IL-33 release via generation of *Staphylococcus* enterotoxins and serine protease-like proteins and promoting eosinophilic migration [16,170,244,245]. *S. aureus* enterotoxin-specific IgE has been associated with elevated mucosal IgE and IL-5 levels and predicts more severe CRS [16]. The outcome of type 2 cytokines/chemokines leads to the recruitment, activation, and improved survival of eosinophils [246]. Mucosal and/or blood eosinophilia and the presence of comorbid asthma are associated with poor outcomes, in terms of quality of life, recurrence of nasal polyps following sinus surgery, and disease severity [153,154,247,248,249]. Other type-2 inflammatory markers, such as IgE, ECP, and IL-5, are also considered predictive markers for recurrence of CRSwNP [22,250].

#### 1.9.2. Mechanism of Non-Type 2 Inflammation

Non-type 2 inflammation is largely characterised by the presence of neutrophils in the nasal mucosa [16,19,214]. Neutrophilic inflammation can be caused by infections or chronic irritation. CRSsNP is typically associated with type 1 inflammation, which is triggered by the release of IL-12 from dendritic cells and macrophages upon antigen exposure [251]. This encourages the differentiation of naive T cells into Th1 cells, as well as the subsequent production of IFN-γ and IL-2 [252]. IFN-γ, in turn, stimulates neutrophil oxidative burst, phagocytosis, and chemotaxis [253]. This pathophysiology is also observed in other inflammatory diseases, such as rheumatoid arthritis, multiple sclerosis, and psoriasis [254].

**Figure 3:**
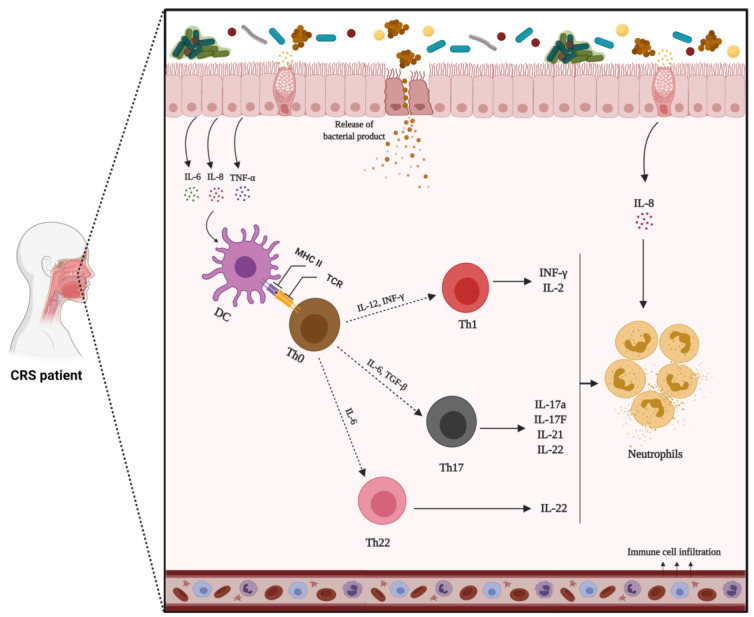
Mechanisms of non-type 2 inflammation in patients with chronic rhinosinusitis without nasal polyps (CRSsNP). In response to environmental stimuli, epithelial cells release inflammatory mediators that induce type 1 (Th1) or 17 (Th17) inflammatory pathways. Th1 and Th17 orchestrate inflammation through the production of IFN-γ, IL-17a, and IL-22. In this pathway, B cell activation and differentiation into plasma cells can produce IgG antibodies that finally lead to neutrophil activation.

The Th17 inflammatory pathway is also associated with CRSsNP. In this pathway, dendritic cells detect pathogens and produce IL-23. This inflammatory mediator, in combination with IL-1β and IL-6, secreted from T cells, induces the expression of IL-22 from different cells. IL-22, in turn, operates synergistically with IL-17, secreted by Th17 cells, and TNF-α to generate various cytokines/chemokines that induce downstream inflammatory effects, including the recruitment of neutrophils [255] (Figure 3).

Th1- and Th17-derived inflammatory mediators have recently been the focus of biologics development in several chronic inflammatory diseases [256]. Investigation into non-type 2 inflammatory disorders lags well behind type 2 inflammatory diseases; yet, no endotype-driven therapeutic agent has been demonstrated to be successful. Since tissue neutrophilia is associated with decreased clinical response to corticosteroids, and further exploration of non-type 2 inflammation is urgently required [257,258].

#### 1.9.3. Mixed Inflammatory Patterns

CRSwNP generally represents the most severe phenotype, with a high recurrence rate and comorbid asthma, and is traditionally characterised by eosinophilic inflammation and a high *S. aureus* colonisation rate [16,150,258]. Nonetheless, CRSsNP has long been regarded as a non-type 2 inflammation, with elevated IFN-γ, TNF-α, IL-17, and IL-21 levels and a predominant neutrophil population [238,239,259]. Even though this dichotomous classification is still valid, CRS endotyping has shed light on the complexity of CRS, with frequent presentations of mixed inflammatory patterns and cellular diversity, emphasising that the current classification cannot solely explain the pathophysiology of CRS [16,166,237,260]. Recent endotype-based studies, which have compared the cytokine profiles of patients with CRSsNP and CRSwNP, have challenged these traditional views [166,261]. Type 2 CRSsNP, similar to CRSwNP, is characterised by tissue eosinophilia with an eosinophil extracellular trap (EET) formation, subepithelial Charcot-Leyden crystal (CLC) deposition, significantly higher rates of asthma and recurrence, and reduced quality of life, compared to CRSsNP without an eosinophilic type 2 response [247,262]. However, the rate of recurrence in type 2 CRSsNP was still significantly lower than in those with CRSwNP [258,263]. On the other hand, several studies over the last decade have observed a mixed eosinophilic-neutrophilic inflammation in CRSwNP patients [201]. Compared to patients with predominantly eosinophilic or neutrophilic CRSwNP, patients with mixed eosinophilic-neutrophilic phenotypes have shown more severe tissue inflammation, with a higher overall inflammatory burden, as measured by computed tomography and the olfactory function, as well as a higher symptom burden [264,265]. Many unanswered questions remain regarding type 2 inflammation in CRSsNP and mixed eosinophilic-neutrophilic inflammation in CRSwNP. The factors driving these responses are not well-understood. For instance, there is no clear reason why CRSsNP and CRSwNP share most of the features of type 2 inflammation, but only the latter is associated with nasal polyps. CRSsNP pathogenesis and its impact on the clinical disease will need to be better defined in future studies [266].

### 1.10. Current Therapeutic Strategies for CRS

CRS patients are primarily treated with standard medical therapy, based on the current consensus guidelines [267]. Nasal irrigation and intranasal steroids are considered the backbones of the pharmacological therapy of CRS, while systemic steroids and antibiotics function as the main relievers during exacerbation onset. Topical steroids have demonstrated beneficial effects in reducing inflammation in CRS patients, with limited side effects. Strong evidence asserts that intranasal corticosteroids efficiently diminish CRS symptoms and polyp development in the nasal cavity [268]. Adjuvant medical therapies in CRS include low-dose macrolides, leukotriene antagonists, topical antibiotics, and oral anti-fungal medicines [269].

Surgery is commonly executed when all attempts at successful medical intervention fail [270]. Diverse surgery options exist, varying from simple polypectomy to the complete removal of the polypoid mucosal tissue from the sinuses [271]. Endoscopic sinus surgery (ESS) is a minimally invasive surgical procedure for CRS that aims to restore sinus ventilation and drainage by opening the main areas and maintaining the sinus mucosa [1]. Furthermore, effective avoidance measures for the target allergen and allergen immunotherapy (AIT) are other well-known strategies [272]. AIT is a highly effective therapeutic approach for allergic disorders, and it induces a long-lasting allergen tolerance by altering the disease course [7].

Numerous potential CRS biomarkers have also been described in the literature but have yet to be clinically validated as indicators of severity or treatment outcome. Eosinophils, IL-4, IL-5, IL-13, and IgE are well-known biomarkers of type 2 inflammation, and some of these are targets for the current biological therapies. Regulatory T cells, IL-25, IL-33, and TSLP are other promising candidates; however, further research is required to validate their role as type 2 biomarkers [273,274]. Despite discovering many potential biomarkers, it is unclear how they can be translated to the bedside [275]. A phase 3 study of duplilumab, a monoclonal antibody (mAb) against IL-4Rα, reported considerable benefits for patients, regardless of the peripheral eosinophil count [276]. The results of phase 2 mepolizumab (anti-IL-5 mAb) found that baseline peripheral eosinophils do not predict improvement in CRSwNP [277]. Dexpramipexole (an anti-eosinophilic synthetic aminobenzothiazole) reduced the number of eosinophils in peripheral blood and nasal polyp tissue; however, the size and symptom scores of nasal polyps did not change [278]. The use of omalizumab (anti-IgE mAb) reduced the size of nasal polyps and improved sinus CT scores but had no significant impact on nasal IgE [279]. The use of duplilumab and omalizumab has been approved by the FDA for difficult-to-treat CRSwNP [280]. However, their effects on patients with mixed inflammatory patterns are unknown, and factors such as long-term safety [281] and cost-effectiveness [282] need to be considered. On the other hand, there are currently no clinical biomarkers indicative of non-type 2 inflammation, which remains an unresolved issue.

Considering that recalcitrant CRS is often found in association with *S. aureus* biofilms, therapeutic strategies targeting this bacterium’s biofilm or virulence factors might be beneficial. Increasing antibiotic resistance among *S. aureus* strains emphasises the necessity for alternative treatments. Anti-virulence treatments, including antibodies, nanoparticles, RNAIII-inhibiting peptides, antimicrobial peptides (AMPs), natural compounds, and vaccines that directly or indirectly neutralise *S. aureus* toxins, have been investigated, and some of them have shown promising effects [118]. However, there is currently no vaccine against *S. aureus*. As mentioned earlier, *S. aureus* secretes a broad spectrum of toxins during the colonisation and infection of the host, making vaccine development challenging. IBT-VO2, as a promising multivalent vaccine, is currently under investigation. α-toxin, PVL, LukS, LukF, LukAB, enterotoxins A and B, and TSST1 toxoids are all included in this vaccine. After completing the encouraging pre-clinical phase, it has recently entered a phase I clinical study [283].

## 2. Conclusions

Substantial advances have been achieved in the understanding of CRS pathogenesis. CRS involves an intricate interaction of infectious, inflammatory, and host factors. It is now obvious that the aetiology of CRS is not as simple as infection by a single pathogenic bacterium. Instead, an imbalance of the sinus microbiome or dysbiosis may play an important role in CRS pathophysiology. Much attention has been focused on *S. aureus*, and whilst it is thought to play an important role in the immunopathogenesis of CRS, the precise role of *S. aureus* and its biofilms in the disease process remains to be investigated. 

With the emergence of bacterial resistance to antibiotics, a huge focus has been placed on CRS endotyping analysis, in order to find alternative therapies, such as biologics, which are considered promising therapeutics for the personalised treatment of CRS patients. The stratification of CRS patients based on endotypes will facilitate the development of specific biomarkers for disease that are associated with each subgroup. Additionally, the use of endotypes in clinical practice in the future is expected to identify the patient groups that will benefit the most from new and existing treatments. Endotype-based therapies might enable physicians to tailor an appropriate therapy regimen with reduced risks, compared to multiple revision surgeries or long-term corticosteroid use. Nevertheless, our limited knowledge of the underlying pathophysiology and lack of objective biologic markers of disease severity and treatment outcomes, relating to the quality of life, remains a barrier; thus, endotype-driven treatment still needs to overcome various challenges before its implementation in daily practice. On the other hand, there is overwhelming evidence that airway-local mucosal B cells drive disease. Thus, B cell function, in the context of human upper airway disease, needs further investigation.

This field of study still lacks an understanding of the association of inflammatory cells/endotypes with the sinus microbiome or bacterial biofilm/virulence factors as important players in CRS pathogenesis. Thus, a more detailed analysis of the disease endotypes in large and highly uniform groups of patients, with robust techniques in association with the microbiome and bacterial products, might provide useful information regarding the disease pathophysiology. The expectation is to eventually translate this knowledge into enhanced patient care. This could result in optimised and individualised treatment for patients suffering from CRS.

## Data Availability

Not applicable.
